# Airway management and pulmonary aspiration during surgical interventions in pregnant women in the 2nd/3rd trimester and immediate postpartum – a retrospective study in a tertiary care university hospital

**DOI:** 10.1186/s12871-024-02551-4

**Published:** 2024-05-03

**Authors:** Charlotte E. Becker, Wolfram Lorenz, Marcelo Gama de Abreu, Thea Koch, Thomas Kiss

**Affiliations:** 1 Department of Anesthesiology and Intensive Care Medicine, University Hospital Carl Gustav Carus, Technische Universität Dresden, Dresden, Germany; 2 Division of Intensive Care and Resuscitation, Department of Anesthesiology, Integrated Hospital Care Institute, Cleveland Clinic, Cleveland, OH United States; 3 Outcomes Research Consortium, Department of Anesthesiology, Integrated Hospital Care Institute, Cleveland Clinic, Cleveland, OH United States; 4 Division of Cardiothoracic Anesthesia, Department of Anesthesiology, Integrated Hospital Care Institute, Cleveland Clinic, Cleveland, OH United States; 5 Department of Anesthesiology, Intensive-, Pain- and Palliative Care Medicine, Radebeul Hospital, Academic Hospital of the Technische Universität Dresden, Heinrich-Zille-Straße 13, 01445 Radebeul, Germany

**Keywords:** Pregnancy, Aspiration pneumonia, Airway management, Laryngeal masks, Postpartum haemorrhage

## Abstract

**Background:**

Pregnancy is associated with an increased risk of pulmonary aspiration during general anaesthesia, but the incidence of this complication is not well defined.

**Methods:**

We performed a retrospective database review in a tertiary care university hospital to determine the incidence of pulmonary aspiration in pregnant patients undergoing endotracheal intubation, with and without Rapid Sequence Induction (RSI), as well as face-mask ventilation and supraglottic airway devices. We included Patients in the 2^nd^ or 3^rd^ trimester of pregnancy and immediate postpartum undergoing surgical procedures. The primary endpoint was the occurrence of pulmonary aspiration.

**Results:**

Data from 2,390 patients undergoing general anaesthesia for cerclage of cervix uteri, manual removal of retained placenta, repair of obstetric laceration, or postpartum bleeding were retrospectively evaluated. A supraglottic airway device or face-mask ventilation was used in 1,425/2,390 (60%) of patients, while 638/2,390 (27%) were intubated. RSI was used in 522/638 (82%) of patients undergoing tracheal intubation, or 522/2,390 (22%) of the entire cohort. In-depth review of the charts, including 54 patients who had been initially classified as “possible pulmonary aspiration" by anaesthetists, revealed that this adverse event did not occur in the cohort.

**Conclusions:**

In conclusion, in this obstetric surgery patient population at risk for pulmonary aspiration, supraglottic airway devices were used in approximately 60% of cases. Yet, no aspiration event was detected with either a supraglottic airway or endotracheal intubation.

**Supplementary Information:**

The online version contains supplementary material available at 10.1186/s12871-024-02551-4.

## Background

The risk of aspiration in pregnant women is poorly defined. Ever since the report of gastric content aspiration by Mendelson, there has been a great fear of aspiration and its consequences during pregnancy. In addition to a potential difficult airway, an increased Body Mass Index (BMI) and the urgency of peripartum procedures, alterations in physiological processes, such as the delay of gastric emptying or a decrease in the lower oesophageal sphincter with consecutive gastroesophageal reflux are said to evoke this risk [1, 2]. Additionally, a reduced functional residual capacity favours rapid desaturation in the phase of apnoea during intubation and lowers the hypoxia threshold after aspiration.

For the above reasons, the thresholds for use of Rapid Sequence Induction (RSI) have been decreasing over time, and may vary considerably. Furthermore, various recommendations have been proposed for RSI based on gestational age: from the 12^th^ week of pregnancy, [3] from the 13^th^ week, [4] from the 18^th^ week, [5] or starting from the 3^rd^ trimester, thus approximately the 27^th^ week of pregnancy, [6] contributing to uncertainty among anaesthesiologists. In fact, even the recommendation against the use of Laryngeal Mask (LMA) for caesarean section in the 3^rd^ trimester of pregnancy was questioned [7]. Currently, there are no societal guidelines or binding recommendations on the use of supraglottic airway devices in pregnancy. At the Dresden University Hospital, supraglottic airway devices have been used in certain situations during pregnancy, based on individual considerations. In view of the impossibility of confirmatory studies in this setting, (e.g. low event rates, preoperative patient information and consent), cases conducted at the Dresden University Hospital in the last 15 years were analysed.

We compared airway management with endotracheal intubation, including RSI versus manual circle system face-mask ventilation and supraglottic airway devices in respect to the occurrence of pulmonary aspiration in pregnant women in the 2^nd^ and 3^rd^ trimester undergoing peripartum surgical procedures in our institution. We hypothesised that the use of face-mask ventilation and supraglottic airway devices during general anaesthesia compared to endotracheal intubation was associated with higher occurrence of pulmonary aspiration.

## Methods

The ethics committee of the Technische Universität Dresden, Germany, approved this study (Reference: BO-EK-98032020, Chairperson PD Dr. med. H. Theilen) on 26 March 2020. The need for written informed consent was waived by the ethics committee of the Technische Universität Dresden, Germany due to retrospective nature of the study.

Inclusion criteria were as follows: procedures following the ICD (International Classification of Diseases, World Health Organization) code O72 (postpartum bleeding) or the surgical code (German modification of the International Classification of Procedures in Medicine, World Health Organization) 5-674 (cerclage of cervix uteri), 5-756 (manual removal of retained placenta) or 5-758 (Repair of obstetric laceration) and anaesthesiologic participation. We excluded patients with incorrect coding of procedures.

We included patients undergoing the above-mentioned procedures as we intended to select those who presented risk factors for pregnancy-associated pulmonary aspiration, such as an enlarged uterus, [8] a decrease in the lower oesophageal sphincter with consecutive gastroesophageal reflux or increased BMI [4, 9].

Data acquisition covered the period from 1/January/2005 to 31/May/2020. All patients were treated in the Department of Gynaecology and Obstetrics at the University Hospital of Dresden, Germany. Approximately 160 anaesthesiologists are employed at the University Hospital, 40% of whom are qualified (anaesthesia specialist level) to work in the delivery room and the corresponding operating room. As risk factors such as enlarged uterus and increased BMI become more pronounced in the later stages of pregnancy, we excluded patients in the 1^st^ trimester. We searched the anaesthesia database of the University Hospital Dresden for the respective charts. The anaesthesia documentation included detailed data on the preoperative, intraoperative, and postoperative course (see Document, Additional file 1, which demonstrates a detailed description).

A digital scan of the original anaesthesia documentation was manually analysed. When necessary, the complete patient record was assessed.

We looked specifically for data regarding airway management including use of manual circle system face-mask ventilation, supraglottic airway devices, tracheal intubation, and regional anaesthesia as follows:

- Preoperative conditions
  - ◦ Weight, Height, Age, ASA (American Society of Anaesthesiologists), Mallampati, Arné/Wilson score, [10, 11] fasting period, urgency classification, patient history and comorbidities, laboratory values, anamnestic features (such as gestation week)
- Anaesthetic induction
  - ◦ Premedication drugs, Cormack and Lehane score, [12] drugs for anaesthesia induction
- Anaesthetic course/Intraoperative values
  - ◦ Standard monitoring values such as blood pressure, heart rate, oxygen saturation, capnometry, duration of interventions, comments, complications, transfusion of blood products, application of drugs, ventilator settings, side effects and complications (see Document, Additional file 1, which demonstrates a detailed AVB number listing) during anaesthesia
- Post-anaesthesia care
  - ◦ Standard monitoring values such as blood pressure, heart rate, oxygen saturation, time spent in the recovery unit, postoperative complications, analgesic and antiemetic drugs, transfusion of blood products

We examined the documents for signs of pulmonary aspiration (primary endpoint) according to the criteria published by Bernardini et al: [13]

- pulmonary aspiration was considered to have occurred if: (1) gastric contents, bilious fluid or other non-respiratory secretion was suctioned from the trachea; or (2) dyspnoea, hypoxia, auscultatory abnormalities and/or new infiltrates on chest X-ray were present after visualization of gastric contents, bilious fluid or other non-respiratory secretion in any part of the endotracheal tube/LMA or the oropharynx.

We also analysed the charts for relevant data on potential pulmonary aspiration. We adapted the criteria used by Ezri et al. to detect pulmonary aspiration [14].

- Evidence of aspiration by the treating anaesthesiologist
- Decrease of oxygen saturation measured by pulse oximetry by more than 5 % from the initial value during anaesthesia
- Need for bronchoscopy in case of clinical suspicion of pulmonary aspiration without detection of bilious secretions or solid particles
- Documentation of laryngo- or bronchospasm with exclusion of other causes than pulmonary aspiration
- Admission to an intensive care unit (ICU) or an intermediate care unit (IMC)
- Continued mechanical ventilation after surgery

Furthermore, post-anaesthesia care was analysed. Patients were classified as stable or unstable in terms of vital parameters, as well as organ function, including breathing, circulation and consciousness by the treating anaesthesiologist (without having defined parameters in advance).

The urgency of surgery was classified as elective, urgent (within 24 hours), emergency (within 2 hours) or vital (immediate). Additionally, we assessed the charts for documentation of difficult airway, which was defined as more than one attempt at intubation or Cormack-Lehane Grade 3 or 4 during direct laryngoscopy [12].

The Strengthening the Reporting of Observational Studies in Epidemiology (STROBE) Statement guidelines was used for reporting results [15].

### Statistics

Data analysis was performed with SPSS (Version 28, IBM, New York, NY, USA). Data is presented in absolute numbers and percentage. Non-normal distributed data is expressed as median and interquartile range. We tested for normal distribution with Kolmogorov-Smirnov and Shapiro-Wilk tests. Non-normal distributed data was assessed with Kruskal-Wallis tests, followed by Dunn’s tests for multiple comparisons. Significance was accepted at alpha = 0.05.

Missing data from the anaesthesia documentation was completed by analysis of the complete medical record.

## Results

**Fig. 1:**
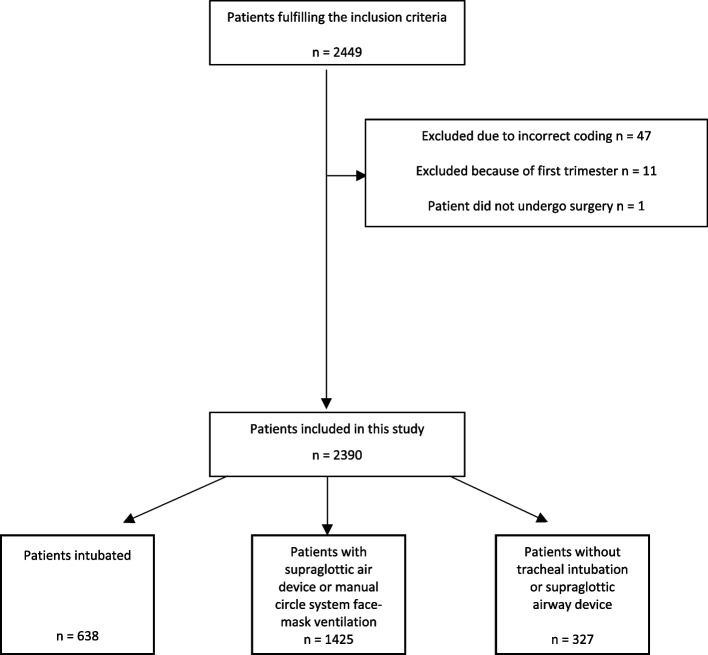
Study flow chart (CONSORT)

A total of 2,449 patients fulfilled the inclusion criteria. We excluded eleven patients because they were in the 1^st^ pregnancy trimester, 47 patients due to incorrect coding of procedures and one patient did not undergo surgery. In total, 2,390 patients were included in the analysis (Fig. 1).

Patients’ characteristics are shown in Table 1. None of them received liquid oral acid aspiration prophylaxis or other premedication (proton pump inhibitors, H2 antagonists) prior to surgery. Of the 2,390 patients, 638 were intubated (26.7%). Among the patients who were intubated, 522/638 (81.8%) were managed with RSI. A supraglottic airway device or manual circle system face-mask was used in 1425 patients (59.6 %). Due to the long observation period of 15 years, it was not possible to determine exactly which generation of laryngeal masks were in use, but given the time frame, we assume that first-generation masks were predominantly used.

**Table 1:** Demographic data of patients undergoing the following procedures: (cerclage of the cervix uteri, removal of retained placenta, suture after perineal tear or any intervention following a postpartal bleeding of patients in the 2^nd^ or 3^rd^ trimester of pregnancy or immediate postpartum

	Total 2390	Intubation 638	Supraglottic/ Mask 1425	Other 327
Age [Years]	31,0 (IQR 7,0)	31,0 (IQR 7,0)	31,0 (IQR 7,0)	31,0 (IQR 7,3)
BMI [kg/m^2^]	25,7 (IQR 6,2)	26,0 (IQR 5,9)	25,0 (IQR 5,9)	27,4 (IQR 6,2)
BMI < 18.5 kg/m^2^ (Underweight)	15 (100)	2 (13,3)	13 (86,7)	0 (0)
BMI 18.5-24.9 kg/m^2^ (Normalweight)	683 (100)	138 (20,2)	487 (71,3)	58 (8,5)
BMI 25-29.9 kg/m^2^ (Overweight)	829 (100)	216 (26,1)	490 (59,1)	123 (14,8)
BMI 30-34.9 kg/m^2^ (Obesity 1°)	362 (100)	86 (23,8)	209 (57,7)	67 (18,5)
BMI 35-39.9 kg/m^2^ (Obesity 2°)	105 (100)	23 (21,9)	60 (57,1)	22 (21,0)
BMI ≥ 40 kg/m^2^ (Obesity 3°)	78 (100)	20 (25,6)	36 (46,2)	22 (28,2)
Missing	318 (100)	154 (48,4)	130 (40,9)	34 (10,7)
Cigarette smoking (currently smoking & ex-smoking)	108 (100)	19 (17,6)	80 (74,1)	9 (8,3)
Substance abuse (use of a drug in amounts or by methods that are harmful)	19 (100)	9 (47,4)	7 (36,8)	3 (15.8)
Gastroesophageal Reflux Disease	45 (100)	14 (31,1)	29 (64,4)	2 (4,4)
Chronic Disease				
Kidney				
Acute Kidney Injury	1 (100)	1 (100)	0 (0)	0 (0)
Chronic Kidney Disease	3 (100)	1 (33,3)	2 (66,7)	0 (0)
Requiring Dialysis	2 (100)	1 (50)	1 (50)	0 (0)
Diabetes	65 (100)	20 (30,8)	28 (43,1)	17 (37,8)
Asthma	85 (100)	13 (15,3)	56 (65,9)	16 (18,8)
Hypothyroidism	229 (100)	43 (18,8)	154 (67,2)	32 (14,0)
Respiratory Infection	4 (100)	2 (50)	2 (50)	0 (0)
Preoperative Anemia				
Hb < 5mmol/l	20 (100)	13 (65)	6 (30)	1 (5)
Hb 5 – 6,3mmol/l	63 (100)	27 (42,9)	32 (50,8)	4 (6,3)
Hb 6,3 – 7,5mmol/l	244 (100)	71 (29,1)	139 (57,0)	34 (13,9)
Time Of Surgery				
During the day	1522 (100)	326 (21,4)	991 (65,1)	205 (13,5)
At night	868 (100)	312 (36,0)	434 (50)	122 (14,0)
Regional Anesthesia				
Yes	442 (100)	36 (8,1)	84 (19,0)	322 (72,9)
No	1948 (100)	602 (30,9)	1341 (68,8)	5 (0,3)

Values are given as Number (percentage of total) or median and interquartile range. *BMI* Body Mass Index, *Hb* Hemoglobin, *IQR* Interquartile range

Independent samples Kruskal-Wallis Test showed that the distribution of Age is the same across categories of Airway Management (p-value 0.208), and also showed a difference in the distribution of BMI across categories of Airway Management (*p*-value <0.001). Post hoc analysis by Dunn’s Test with adjusted significance level results were: Supraglottic vs. ITN *p*=0.002; Supraglottic vs. Other p<0.001; ITN vs. Other *p*<0.001

Regional anaesthesia, with or without general anaesthesia, was used in 442/2,390 patients (18.5%), while 327/2,390 (13.7%) did not receive neither tracheal intubation, nor a supraglottic airway device.

No signs of pulmonary aspiration could be identified in our study population. There were 64 documented events of possible aspiration in 54 patients (some patients had more than one event). In one of the patients, bronchoscopy was performed during anaesthesia. Among the remaining, 6 patients were transferred intubated after surgery, 24 patients were transferred to ICU, 3 patients went to IMC and 30 patients had a drop in SpO_2_ during anaesthesia of more than 5 % compared to the initial value. Characteristics of these patients, including airway management, are summarised in Table 2. (see Table S1 Additional file 1 for a more detailed listing)

**Table 2:** Overview of 64 events (in 54 patients) that fulfilled criteria for suggesting pulmonary aspiration and the corresponding airway management

Reason for suggesting pulmonary aspiration	Number of events	Airway management
Bronchoscopy	1	Mask, then ITN
Transferred intubated	6	4 ITN 1 LMA, then ITN 1 Mask, then ITN and Bronchoscopy
Transferred to ICU	24	20 ITN 1 LMA 1 LMA, then ITN 1 Spinal Block, then ITN 1 Mask, then ITN and Bronchoscopy
Transferred to IMC	3	1 Mask, then ITN 1 LMA, then ITN 1 ITN
Drop in SpO_2_ by more than 5 % from the initial value	30	14 Mask 3 LMA 1 Spinal block, then LMA 10 ITN 1 Mask, then LMA, then ITN 1 Mask then ITN and Bronchoscopy

*Mask* Manual circle system face-mask ventilation, *ETT* Endotracheal tube, *insufflation* oxygen insufflation via nasal cannula or non-rebreather mask, *LMA* Laryngeal mask, *ICU* Intensive care unit, *IMC* Intermediate care unit, *SpO2* oxygen saturation measured by pulse oximetry

In the one patient who underwent bronchoscopy, aspiration was not confirmed. In patients who were transferred to ICU or IMC, medical charts also did not code aspiration events. In 30 cases, an absolute SpO_2_ difference of > 5 % between the initial value and the minimum value during anaesthesia was documented. In 26 out of those 30 cases, there was no further evidence of aspiration, or the anaesthesiologist even explicitly excluded its occurrence. In two patients, the reason for the drop in saturation was attributed to hemodynamic instability. In one patient, a resident anaesthesiologist described a difficult manual circle system face-mask ventilation, which could be solved by the staff anaesthesiologist. The patient, in whom the bronchoscopy was performed, also fulfilled the criteria of saturation drop greater than 5 %.

Details of all procedures requiring general anaesthesia are listed in Table 3.

**Table 3:** Procedures requiring anesthesia and characteristics of intervention

	Total 2390 (100 %)		Intubation 638 (26.7 %)		Supraglottic 1425 (59.6 %)		Other 327 (13.7 %)	
ASA	All patients	PPA	All patients	PPA	All patients	PPA	All patients	PPA
1	908 (100 %)	15	202 (22.2 %)	4	604 (66.5 %)	11	102 (11.2 %)	0
2	1183 (100 %)	23	331 (28 %)	11	668 (56.5 %)	11	184 (15.5 %)	1
3	98 (100 %)	5	46 (47 %)	4	41 (41.8 %)	0	11 (11.2 %)	1
4	17 (100 %)	6	12 (70.6 %)	5	5 (29.4 %)	1	0 (0 %)	0
5	1 (100 %)	1	1 (100 %)	1	0 (0 %)	0	0 (0 %)	0
Missing	183 (100 %)	4	46 (25.1 %)	4	107 (58.5 %)	0	30 (16.4 %)	0
Procedures								
Cervical cerclage	349 (100 %)	5	17 (4.9 %)	1	295 (84.5 %)	4	37 (10.6 %)	0
Removal of ret. placenta	1418 (100 %)	29	459 (32.4 %)	15	781 (55 %)	14	178 (12.6 %)	0
Perineal repair	77 (100 %)	1	23 (29.9 %)	0	38 (49.4 %)	1	16 (20.7 %)	0
Placenta removal+suture	357 (100 %)	0	107 (30 %)	0	185 (51.8 %)	0	65 (18.2 %)	0
Other (Postpartum bleeding)	189 (100 %)	21	32 (16.9 %)	15	126 (66.7 %)	4	31 (16.4 %)	2
Fasting								
>6 hours	1256 (100 %)	26	159 (12.7 %)	7	990 (78.8 %)	18	107 (8.5 %)	1
<6 hours	189 (100 %)	5	82 (43.4 %)	3	87 (46.0 %)	2	20 (10.6 %)	0
<4 hours	162 (100 %)	4	109 (64.9 %)	4	28 (17 %)	0	25 (15.4 %)	0
<2 hours	171 (100 %)	5	111 (65.3 %)	4	29 (16.2 %)	0	31 (18.1 %)	1
Unknown	207 (100 %)	5	88 (42.5 %)	5	55 (26.6 %)	0	64 (30.9 %)	0
Missing	405 (100 %)	9	89 (22 %)	6	236 (58.3 %)	3	80 (19.7 %)	0
Urgency								
Elective	741 (100 %)	12	93 (12.6 %)	1	556 (75 %)	9	92 (12.4 %)	2
Urgent (within 24 h)	733 (100 %)	13	161 (22 %)	6	387 (52.8 %)	7	185 (25.2 %)	0
Emergency (within 2 h)	703 (100 %)	18	284 (40.4 %)	12	388 (55.2 %)	6	31 (4.4 %)	0
Vital	75 (100 %)	11	58 (77.3 %)	10	16 (21.3 %)	1	1 (1.3 %)	0
Missing	138 (100 %)	0	42 (30.4 %)	0	78 (56.5 %)	0	18 (13 %)	0

Airway management of patients undergoing the following procedures: (cerclage of the cervix uteri, removal of retained placenta, suture after perineal tear or any intervention following a postpartal bleeding of patients in the 2^nd^ or 3^rd^ trimester of pregnancy or immediate postpartum. Other patients includes all patients without tracheal intubation or supraglottic airway device. The event rate of Potential Pulmonary Aspiration (PPA) is shown as absolute number(percentage), out of 64 documented events that have been recorded. Values are given as Number (percentage). *ASA* American Society of Anesthesiology. Potential pulmonary aspiration = PPA. h = hours

With respect to a difficult airway, Wilson score [11] has been used until 2011, thereafter the newer Arné score [10] was used. A Wilson score greater than 2, was found in 8 out of 606 (1.3 %) patients. Two patients had an Arné score greater than 11 in 976 (0.2 %) patients. Score documentation was missing in 808 out of 2390 patients (33.8 %).

In 638 patients that were intubated, difficult intubation was described in six cases, including one case where intubation failed and a supraglottic airway device was used. Fibreoptics or video-laryngoscopy were not used in any patient**.**

The Cormack and Lehane score was analysed in 605 patients that were intubated, in 33 cases the score was not documented. In 548 cases (90.6 % out of 605) the Cormack and Lehane score was 1, two difficult intubations (0.4 % out of 548) and one failed intubation (0,2 % out of 548) were reported in this group. A Cormack and Lehane score of 2 was recorded in 52 patients (8.6 % out of 605), one difficult intubation (1.9 % out of 52) was reported. A Cormack and Lehane score of 3 was documented in four cases (0.7 % out of 605), two difficult intubations (50 % out of 4) were reported. In one patient (0.2 % out of 605) a Cormack and Lehane score of 4 was described, a difficult intubation was not documented separately. All difficult/failed intubations have initially been performed as RSI using Succinylcholine.

Emergency surgery, including cases with immediate and 2-hour priority, was performed in 778 patients (32.5 %). Fasting was less than 6 hours for food and 2 hours for fluids in 522 of all patients (21.9 %), in 612 patients (25.6 %) fasting period is either unknown/unclear or not documented. There were 868 (36.3 %) patients operated during the night (8 pm-6 am).

The postoperative status of patients receiving intubation was judged as stable in 569/638 (89.2 %) patients and unstable in 11/638 (1.7 %) patients. Data regarding the postoperative status was missing for 58/638 patients (9.1 %). In 20/638 cases (3.1%) admission to the ICU was necessary, but not all these patients were unstable.

The postoperative status of patients managed with a supraglottic airway device and manual circle system face-mask ventilation was described as stable in 1322/1,425 cases (92.8 %) and unstable in three cases (0.2 %). However, in 100/1,425 cases (7.0 %) the corresponding data was missing. In 3/1,425 (0.2 %) cases after manual circle system face-mask ventilation or use of a supraglottic airway device, a transfer to ICU was necessary.

The postoperative status of one patient without intubation or supraglottic airway support/manual circulatory ventilation was described as unstable (0.3 %) and the patient transferred to ICU after surgery.

## Discussion

The data of the present study shows that supraglottic airway devices are common in clinical practice and are used in the majority of cases, not only in low-risk cases, but to a considerable extent even in vital interventions. A comparison of patients who received supraglottic airway management versus those who were intubated shows that intubation was preferred in non-fasting patients, in patients with ASA 4 classification, in patients undergoing urgent procedures, as well as in patients undergoing highly complex surgical interventions. This leaves room for the assumption that tracheal intubation still seems to be the method of choice and indeed the standard in high-risk cases. LMA and bag mask ventilation was predominantly used in obese patients and smokers, as well as those with history of gastroesophageal reflux disease, diabetes, asthma and hypothyroidism. Although the use of laryngeals masks, including safety and efficacy in pregnant women has been addressed in more recent studies, those reports were limited to caesarean sections [7, 16].

The postoperative status of patients receiving intubation was judged as unstable in 1.7 %. In those managed with a supraglottic airway device or with bag mask ventilation, 0.2 % were judged as unstable. Subsequently, the reason for this assessment cannot be determined any more. However, it seems likely that unstable patients are preferentially managed by tracheal intubation.

We intended to select patients who present risk factors for pregnancy associated pulmonary aspiration, such as an enlarged uterus, a decrease in the lower oesophageal sphincter with consecutive gastroesophageal reflux or increased BMI. However, several studies could not confirm the BMI as an independent risk factor for pulmonary aspiration [17, 18]. Interestingly, obese patients were managed predominantly with LMA and bag mask ventilation, although higher grades of obesity are said to be associated with a higher risk of aspiration. Not only elective but also emergency procedures that are believed to have a higher risk of aspiration were considered [9, 17]. In our study, 32.5 % of all interventions were emergency procedures. A selection of other factors generally thought to increase the risk did not lead to aspiration either: At night, 868 (36.3 %) operations were performed. Out of the 2390 patients, 4.8 % were classified as risk class ASA 3 or higher. Overall, it was found that neither pregnancy alone, nor in combination with one or more risk factors, promoted aspiration in our retrospective analysis.

In the literature on pulmonary aspiration in pregnant women, incidences range from 0.01 – 0.23 %, [19, 20] more recent data describes an incidence of 0.022 % for pulmonary aspiration during pregnancy or immediately postpartum in association with general anaesthesia [21]. Our sample size of 2390 patients was not high enough to safely detect a single case of pulmonary aspiration. When referring to data that has been recorded more than 25 years ago, one must keep in mind that technical achievements, growth in knowledge and training in the field of anaesthesia have been further developed. Second-generation supraglottic airway devices that have better, although not complete, protection against aspiration are now recommended as rescue airway devices after failed tracheal intubation, particularly in patients at increased risk of aspiration [22].

It is difficult to compare studies investigating the incidence of pulmonary aspiration in an obstetric surgical population, since the definition of pulmonary aspiration is inconsistent, as are other factors, such as patient populations and interventions considered, and the level of training of the anaesthesiologist performing the procedure. Several authors have already referred to the ambiguous definition of pulmonary aspiration [17, 23].

Difficult intubation is another independent risk factor for aspiration [4, 24]. In our study population, we found 3 difficult intubations and one failed intubation (finally managed with LMA) within the Cormack and Lehane score 1 & 2 group and 5 patients with Cormack and Lehane score 3 & 4.

We found an incidence of difficult/failed intubation of 1.4% (9 out of 639) in our study population. Difficult intubation using Wilson and Arné scoring was predicted in 10 out of 1582 cases (0.6%). However, not all anaesthesiologists seem to agree that a Cormack and Lehane score of 3 should be considered as being difficult to intubate [25].

Existing data regarding difficult intubation is poor because most studies examine the incidence of failed intubation [25, 26]. Values in the range from 3.3 % - 7.9 % are documented for the incidence of difficult intubation in obstetric anaesthesia, but these do not coincide with those of the present work, where an incidence of 1.4% has been found [25, 27].

In general, it is said that obstetric patients are more difficult to intubate than general surgery patients [28, 29]. The definitions used for airway problems are inconsistent and potentially misleading. Definitions include failure to intubate, three or more laryngoscopies, four or more attempts to pass the endotracheal tube or longer duration of endotracheal placement (>10 minutes), or a poor view of the vocal cords via direct laryngoscopy following anaesthesia induction [30]. Due to inconsistencies in defining difficult intubation, comparability of our data with other studies seems to be hardly possible [25, 26]. The incidence of difficult intubation in our study is lower than those published by the mentioned authors.

The incidence of failed intubations in the field of obstetric anaesthesia is described as being between 0.08 - 0.45 % and 0.1 % - 0.47 % in the general surgical population [26, 30, 31]. In this retrospective study, the rate of failed intubations is 0.16 % (1 out of 638) and thus in the range of the discussed values in the obstetric population.

When comparing the number of potential pulmonary aspiration events in relation to specific surgical characteristics, the highest risk is found in ASA 2 patients that are fasting for more than 6 hours and undergo a removal of retained placenta procedure in the setting of an emergency surgery. However, the absolute figures are put into perspective when the frequency distribution is considered, so that no intervention-specific risk profile can be identified.

Despite the fact that our data was collected in a single centre, it might have implications. The risk of aspiration during general anaesthesia for obstetric procedures managed without tracheal intubation in our study population was not higher than that reported in the literature. In the majority of our patients, airway management was safely performed with supraglottic airway devices. Especially when it comes to a "cannot intubate" situation, one should be aware that in our work many cases could be treated with a supraglottic airway without complications. Therefore, our data might support the concept that intubation should not be forced, especially in pregnant women, even if the physiological changes of pregnancy are already present. In our opinion, the gestational age should be rated to a smaller amount in deciding whether RSI induction is indicated. Rather, clinical assessments and anaesthetist’s experience level should be given greater consideration. The necessity of endotracheal intubation to secure the airway in pregnant women [14, 32, 33] and the procedure of RSI [32, 34] has been questioned by different authors.

Our study has several limitations. First, as it is a retrospective analysis our results are prone to misclassification bias. Data was manually extracted from the anaesthesia database with the greatest possible diligence, but definitions and procedures were not standardised a priori.

Second, since this was a single-centre study, caution is advised when extrapolating our findings to other centres. Third, the sample size was relatively small compared to reports, [16, 19–21] which may increase the risk of selection bias. Since the incidence of pulmonary aspiration is lower than 1:2000, [17, 21, 35] we cannot completely rule out that absence of this adverse event is attributable to chance. However, this limitation could be overcome through the use of national databases or by collaborating in a multicentre setting to retrospectively examine aspiration complications.

## Conclusion

In conclusion, in this obstetric surgery patient population at risk for pulmonary aspiration, supraglottic airway devices were used in approximately 60% of cases. Yet, no aspiration event was detected with either a supraglottic airway or endotracheal intubation.

### Supplementary Information

**Supplementary Material 1.**

## Data Availability

The datasets used and/or analysed during the current study are available from the corresponsing author on reasonable request.

## Abbreviations

RSI: Rapid Sequence Induction or Rapid Sequence Intubation
BMI: Body Mass Index
LMA: Laryngeal Mask
ICD: International Classification of Diseases, World Health Organization
ASA: American Society of Anaesthesiologists
AVB: Numbers are part of a uniform documentation data set in Germany, which maps and ensures quality assurance and enables a comparability of different centers (see Additional file 1 for more information)
IMC: Intermediate Care Unit (invasive hemodynamic monitoring and therapy, noninvasive ventilation)
ICU: Intensive Care Unit (ICU capabilities plus invasive ventilation, haemodialysis)
STROBE: Strengthening the Reporting of Observational Studies in Epidemiology
