# Supplementary material for: Airway management and pulmonary aspiration during surgical interventions in pregnant women in the 2nd/3rd trimester and immediate postpartum – a retrospective study in a tertiary care university hospital
Source: BMC Anesthesiol. 2024 May 3;24:166. doi: 10.1186/s12871-024-02551-4 (PMC11067247; doi:10.1186/s12871-024-02551-4)
Supplement: Supplementary file 1 — Supplementary Material 1. [file 12871_2024_2551_MOESM1_ESM.docx]

Airway management and pulmonary aspiration during surgical interventions in pregnant women in the 2nd/3rd trimester and immediate postpartum –

A retrospective study in a tertiary care university hospital

**Supplemental Digital Content**

**Anaesthesia protocol**

The anaesthesia protocol at the University Hospital Dresden incorporates the following variables/information:

Preoperative situation:

Checkboxes:

- Gender, age, urgency, fasting, patient being ventilated at time of preoperative visit, wound classification in case of acute or chronic wounds, previous anaesthesia and its complications, weight, height, ASA Score, Mallampati score, Arné score (Wilson Score until 2011), allergies, other anaesthesia relevant risks (substance abuse/cigarette smoking, infectious disease, difficult airway, pregnancy, aspiration risk), long-term medication, general patient condition, further information concerning chronic diseases such as coronary disease, NYHA score in case of heart failure, hypertensive disease, respiratory disease, neurologic disease, metabolic disease, muscle disease, anatomic disorders, hemophiliac & coagulation disorder, vascular system disease, liver and kidney function/disease, hematologic condition, dental status, if applicable ECG or chest x-ray abnormalities, acid base status/electrolyte values and if necessary also other laboratory values. Date/location/time/duration of preoperative visit and name and signature of anaesthesiologist, type of duty during preoperative visit (regular or on-call service), cardiac frequency, blood pressure, temperature, admission type (inpatient or outpatient care).
- Concerning the planned procedure: current diagnosis and planned surgical procedure, recurring anaesthesia event, emergency procedure, preoperative sedative prescription, blood products to be provided, blood group.

Free text:

- Further patient history and special remarks, preoperative medical order.

Induction and intraoperative course:

Checkboxes:

- Sedative effect of premedication drug, Cormack and Lehane Score, treating surgical department, date of surgery, anaesthesia workplace, extent of monitoring, airway management, intraoperative body positioning, classification of surgery (surgery, polytrauma emergency treatment, diagnostic procedure, pain therapy, obstetrics) use of local anaesthetics, type of used anaesthetics, use of antagonist and neuromuscular blocking drugs as well as other drugs, use of specific equipment or procedures such as gastric tube, cellsaver, bronchoscopy, videolaryngoscopy, resuscitation or other more specific treatment
- Anaesthesia induction technique, anaesthesia maintenance technique, type of regional anaesthesia, use of ultrasound, type of anaesthetic system, used ventilatory mode, intravenous lines (puncture site, diameter of catheter, use of ultrasound), type of infusion (crystalloid, colloid, blood products type and quantity, use of cellsaver), type of duty during anaesthesia (regular or on-call service), patient condition after surgery (stable, unstable, spontaneous breathing/intubation, circulation, vigilance, pain), handling ward after surgery (normal ward, intermediate care, intensive care unit or else).
- Numbers: ICD 10 diagnosis code, OPS code, staff number of involved physicians and nurses, amount of fluid excretion (diuresis, blood loss, other fluid losses), time variables (presence of anaesthesia, anaesthesia induction, start of surgery, end of surgery, end of anaesthesia, end of anaesthesia presence), perioperative AVB* (see separate note), date/time/signature.

Free text:

- diagnosis, procedure, name of handling surgeon, name of handling anaesthesiologist, room number of operating room, date of surgery, graphical representation of anaesthesia course with 5-minute resolution (blood pressure, heart frequency, oxygen saturation, temperature, ECG, blood loss, diuresis, graphical marking of anaesthesia presence, induction and begin/end of anaesthesia, intubation and extubation, incision/suture, begin/end of surgical measures), all used drugs, infusion or transfusion, oxygen, ventilator settings/gas exchange variables.
- detailed description of airway management and intubation anatomy as well as patient condition at time of extubation (pain, protective reflexes, vigilance, circulation), if necessary, details concerning the surgical course, if applicable, description of complications or problems during induction or intraoperative.

Recovery room:

Checkboxes:

- start/end of recovery room, type of service, recovery room location (several available), postoperative AVB* (see separate note), monitoring with 5-minute resolution (blood pressure, pulse, oxygen saturation, medication, transfusions, temperature, drainage quantities, diuresis), special measures (patient warm-up, postoperative ventilation, transfusion, reintubation, cleaning after vomiting, gastric tube, etc.), discomfort assessment (nausea, vomiting, shivering, hypothermia, pain, awareness), postoperative pain management, condition at transfer (breathing stable/unstable, circulation stable/unstable, vigilance stable/unstable), transfer destination (ICU, IMC, normal ward, labor ward, discharge, death)

Free text:

- treating physician and nurse, nurse's qualification, remarks such as complications or other anomalies, physician directions for the ward (infusion, monitoring, lab controls, nutritional supply, pain management), date/time/signature.

Laboratory values

- incl. point of care measurements such as blood gas analysis, all laboratory values ascertained during the operation or in the recovery room, documentation of transfusion (labels of the blood units, charge documentation), bedside test label

Also attached is a detailed scoring sheet of the Arné score for evaluation of a difficult airway and a list of the AVB.

**AVB numbers**

AVB numbers are part of a uniform documentation data set in Germany, which maps and ensures quality assurance and enables a comparability of different centers. AVB is an adverse event that occurs during anaesthesia and gives rise to intervention and causes morbidity or mortality for the patient or would have or could have meant morbidity or mortality for the patient without intervention. It is a code, which is defined with respect to AVB type, AVB severity and AVB time (only two periods).” ^1^

Regarding the AVB type there are 9 subgroups respiratory/airway/lung/thorax events, cardiovascular events, general reactions, laboratory changes, central nervous system events, events during regional anaesthesia, conditions concerning medical devices, damages to the patient’s soft tissue and other problems (surgical problems or organizational procedures that put the patient at risk). In terms of severity, a distinction is made between whether the problem requires a prolonged stay in the recovery room and/or a special follow-up in the general ward or whether the problem cannot be solved satisfactorily in the recovery room and requires a transfer to the intensive care unit or recovery ward, or whether it results in the patient's death. Concerning the time of the AVB it must be chosen between an intraoperative or a postoperative event.^1^

**References**

1. Heinrichs W, Blumrich W, Deil S, Freitag M, Kutz N, Lüdtke I, Röhrig R, Streuf R: Aktualisierter Datensatz zur Durchführung der externen Qualitätssicherung in der Anästhesie Core data set anaesthesia 3.0 / 2010 – Updated data set for external quality control in anaesthesia. Anästh Intensiv 23

Table S1: Overview of the 57 patients that fulfilled criteria for suggesting pulmonary aspiration and the corresponding airway management.

| Patient number | Events | Airway |
| --- | --- | --- |
| 1 | Bronchoscopy  Transferred to ICU  Transferred intubated  Drop in SpO_2_ by more than 5 % from the initial value | Mask, then ITN |
| 2 | Drop in SpO_2_ by more than 5 % from the initial value | Mask, then LMA, then ITN |
| 3 | Drop in SpO_2_ by more than 5 % from the initial value | Mask |
| 4 | Drop in SpO_2_ by more than 5 % from the initial value | Mask |
| 5 | Drop in SpO_2_ by more than 5 % from the initial value | Mask |
| 6 | Drop in SpO_2_ by more than 5 % from the initial value | Mask |
| 7 | Drop in SpO_2_ by more than 5 % from the initial value | Mask |
| 8 | Drop in SpO_2_ by more than 5 % from the initial value | Mask |
| 9 | Drop in SpO_2_ by more than 5 % from the initial value | Mask |
| 10 | Drop in SpO_2_ by more than 5 % from the initial value | Mask |
| 11 | Drop in SpO_2_ by more than 5 % from the initial value | Mask |
| 12 | Drop in SpO_2_ by more than 5 % from the initial value | Mask |
| 13 | Drop in SpO_2_ by more than 5 % from the initial value | Mask |
| 14 | Drop in SpO_2_ by more than 5 % from the initial value | Mask |
| 15 | Drop in SpO_2_ by more than 5 % from the initial value | Mask |
| 16 | Drop in SpO_2_ by more than 5 % from the initial value | Mask |
| 17 | Drop in SpO_2_ by more than 5 % from the initial value | Mask |
| 18 | Drop in SpO_2_ by more than 5 % from the initial value | Mask |
| 19 | Drop in SpO_2_ by more than 5 % from the initial value | LMA |
| 20 | Drop in SpO_2_ by more than 5 % from the initial value | LMA |
| 21 | Drop in SpO_2_ by more than 5 % from the initial value | LMA |
| 22 | Drop in SpO_2_ by more than 5 % from the initial value | LMA |
| 23 | Drop in SpO_2_ by more than 5 % from the initial value | LMA |
| 24 | Drop in SpO_2_ by more than 5 % from the initial value  Transferred intubated  Transferred to ICU | ITN |
| 25 | Drop in SpO_2_ by more than 5 % from the initial value  Transferred to ICU | ITN |
| 26 | Drop in SpO_2_ by more than 5 % from the initial value | ITN |
| 27 | Drop in SpO_2_ by more than 5 % from the initial value | ITN |
| 28 | Drop in SpO_2_ by more than 5 % from the initial value | ITN |
| 29 | Drop in SpO_2_ by more than 5 % from the initial value | ITN |
| 30 | Drop in SpO_2_ by more than 5 % from the initial value | ITN |
| 31 | Drop in SpO_2_ by more than 5 % from the initial value | ITN |
| 32 | Drop in SpO_2_ by more than 5 % from the initial value | ITN |
| 33 | Drop in SpO_2_ by more than 5 % from the initial value | ITN |
| 34 | Transferred intubated  Transferred to IMC | LMA, then ITN |
| 35 | Transferred intubated  Transferred to IMC | ITN |
| 36 | Transferred intubated  Transferred to ICU | ITN |
| 37 | Transferred intubated  Transferred to ICU | ITN |
| 38 | Transferred to IMC | LMA, then ITN |
| 39 | Transferred to ICU | LMA |
| 40 | Transferred to ICU | LMA, then ITN |
| 41 | Transferred to ICU | Insufflation, then ITN |
| 42 | Transferred to ICU | ITN |
| 43 | Transferred to ICU | ITN |
| 44 | Transferred to ICU | ITN |
| 45 | Transferred to ICU | ITN |
| 46 | Transferred to ICU | ITN |
| 47 | Transferred to ICU | ITN |
| 48 | Transferred to ICU | ITN |
| 49 | Transferred to ICU | ITN |
| 50 | Transferred to ICU | ITN |
| 51 | Transferred to ICU | ITN |
| 52 | Transferred to ICU | ITN |
| 53 | Transferred to ICU | ITN |
| 54 | Transferred to ICU | ITN |
| 55 | Transferred to ICU | ITN |
| 56 | Transferred to ICU | ITN |
| 57 | Transferred to ICU | Insufflation, then ITN |

*Mask=* *manual circle system face-mask ventilation; ITN=intubation; insufflation= oxygen insufflation via nasal cannula or non-rebreather mask; LMA=laryngeal mask; ICU=intensive care unit; IMC=intermediate care unit; SpO2=oxygen saturation measured by pulse oximetry*

**List of abbreviations**

LMA Laryngeal Mask

ICD International Classification of Diseases, World Health Organization

ASA American Society of Anesthesiologists

AVB numbers are part of a uniform documentation data set in Germany, which maps and ensures quality assurance and enables a comparability of different centers (see Additional File 1 for more information)

ICU Intensive Care Unit

IMC Intermediate Care Unit

ITN endotracheal Intubation

SpO_2_ Oxygen saturation measured by pulse oximetry
